# Co-infection of *Mycoplasma gallisepticum* and *Escherichia coli* Triggers Inflammatory Injury Involving the IL-17 Signaling Pathway

**DOI:** 10.3389/fmicb.2019.02615

**Published:** 2019-11-15

**Authors:** Zhiyong Wu, Liangjun Ding, Jiaxin Bao, Yuhao Liu, Qiaomei Zhang, Jian Wang, Rui Li, Muhammad Ishfaq, Jichang Li

**Affiliations:** ^1^College of Veterinary Medicine, Northeast Agricultural University, Harbin, China; ^2^College of Life Sciences, Northeast Agricultural University, Harbin, China; ^3^Heilongjiang Key Laboratory for Animal Disease Control and Pharmaceutical Development, College of Veterinary Medicine, Northeast Agricultural University, Harbin, China

**Keywords:** *Mycoplasma gallisepticum*, *Escherichia coli*, co-infection, IL-17 signaling, inflammatory chemokine, RNA-seq

## Abstract

*Mycoplasma gallisepticum* and *Escherichia coli* are well known respiratory disease-inducing pathogens. Previous studies have reported that co-infection by MG and E.coli causes significant economic loss in the poultry industry. In order to assess the respiratory toxicity of co-infection in chicken lung, we established a co-infection model to investigate changes in the inflammatory cytokines, lung tissue structure, and transcriptome profiles of chicken lung. The results showed that co-infection caused a wider range of immune damage and more severe tissue lesions than single-pathogen infection. Differentially expressed gene (DEG) analysis indicated that 3,115/1,498/1,075 genes were significantly expressed among the three infection groups, respectively. Gene ontology and KEGG analysis showed genes enriched in response to immune response, cytokine-cytokine receptor interaction, and inflammation-related signaling pathways. Among these pathways, IL-17 signaling was found to be significantly enriched only in co-infection. The expression of IL-17C, CIKS, TRAF6, NFκB, C/EBPβ, and inflammatory chemokines were significantly up-regulated in response to co-infection. Taken together, we concluded that co-infection increased the expression of inflammatory chemokines in lungs through IL-17 signaling, leading to cilia loss and excessive mucus secretion. These results provide new insights into co-infection and reveal target proteins for drug therapy.

## Introduction

Respiratory infections are a common cause of increased mortality rates and have caused huge economic losses to the poultry industry worldwide (Sid et al., 2015). The pathogens are mainly *Mycoplasma gallisepticum* (*MG*), *Mycoplasma synoviae* (*MS*), *Avian influenza virus* (*AIV*), *Infectious bronchitis virus* (*IBV*), and *Escherichia coli* (*E. coli*) (Dhillon and Kibenge, 1987; Hutchinson, 2018). Among these pathogens, *MG* is often associated with co-infection outbreaks of other pathogens due to a down-regulation of the host immune response by *MG* infection. Stipkovits et al. (2012), Xiao et al. (2015), Sid et al. (2016). In recent years, the threat of co-infection has grown with the expansion of intensive farming. However, there are few reports on co-infection with *MG* and *E. coli*.

Previous reports demonstrated that viral infection skews the antibacterial immune function at the respiratory epithelium (Melvin and Bomberger, 2016; Umar et al., 2018). A review also summarized the effects of co-infection as an altered host cytokine and/or inflammatory environment, including increased or decreased cytokine production or disruption of signaling and responses to cytokines (Stelekati and Wherry, 2012). The mechanisms by which viruses predispose to secondary bacterial infection are damage to airway epithelium, virus-induced type I interferon, enhanced production of inflammatory mediators, etc. (Stelekati and Wherry, 2012; Bakaletz, 2017). Therefore, we focus on immune-related signaling pathways and inflammatory mediators to explore the mechanism of co-infection of *MG* and *E. coli*.

A previous study reported the chicken tracheal response to virulent *MG* R_*low*_ on the basis of transcriptome sequencing (Beaudet et al., 2017). Similarly, the anti-viral genes were shown via the RNA-seq method to be up-regulated in response to infectious bursal disease virus (IBDV) (Smith et al., 2015). In addition, transcriptome analysis revealed inflammatory injury of chicken trachea involving the oxidative stress-mediated FOS/IL8 signaling pathway (Chen et al., 2019). The application of transcriptomics has been widely used as an initial step in demonstrating the harmful effects during infection. Here, the method of RNA-seq was used to find the target genes and related signaling pathways involved in the co-infection (*MG* and *E. coli*) and their underlying mechanisms.

Respiratory disease-induced lung damage is associated with alterations in inflammatory cytokines and related pathways (Melvin and Bomberger, 2016). For instance, NOD-like receptor ligands, IRF1 and STAT1 (Feng et al., 2016), C/EBPβ and CXCL8 (Huang et al., 2017), and IL-17 (Kurai et al., 2013; Jungnickel et al., 2017) are commonly reported. Therefore, we constructed an *in vivo* model of co-infection with *E. coli* and *MG*. Chicken lung tissues were collected for morphological observation and RNA-seq. Then, we examined the *in vivo* mRNA and protein expression of related immune-response genes and cytokines during early infection. Our findings extended the current understanding of the host immune-response mechanism on co-infection and laid a foundation for targeted drug therapy.

## Materials and Methods

### Animals

Eighty (1-day-old) commercial Leghorn chickens were obtained from Chia Chau Chicken Farm (Harbin, Heilongjiang, China) and were assigned randomly to four groups, namely the control group, co-infection group, *MG* group, and *E. coli* group (20 chickens per group). The chickens were in a healthy condition, *MG* and *E. coli* (O78)-free, did not undergo vaccination, and were raised to the 10th day in four separate environmentally controlled chambers (named A, B, C, and D).

### Mycoplasma Strains and Bacteria

The *MG* strain R_*low*_ was obtained from the Harbin Institute of Veterinary Medicine, Chinese Academy of Agricultural Sciences. The mycoplasma were cultured at 37°C in modified Hayflicks medium supplemented with 20% FBS (Gibco BRL) (Gaithersburg, MD, United States), 10% freshly prepared yeast extract, 0.05% penicillin, 0.05% thallium acetate, and 0.1% nicotinamide adenine dinucleotide (NAD). *MG* in its mid-exponential phase, as indicated by the color change of phenol red dye from red to orange, was used to challenge chickens at the density of 1 × 10^9^ CCU/ml (color change unit per milliliter) in the culture medium. The detection of the density of *MG* was performed as described previously (Lu et al., 2017).

*Escherichia coli* O78 was isolated from chickens infected with colibacillosis in our laboratory and cultured in Nutrient Broth (Beijing Aoboxing BIO-TECH Co., Ltd.). The concentration of *E. coli* was adjusted to 10^9^ CFU/ml before infection.

### Chicken Infection

1. Control group (A): Fed in the same environment and kept until the end of the experiments.
2. Co-infection group (B): 0.2 ml of *MG* medium (1 × 10^9^ CCU/ml) was injected into the left caudal thoracic air sac at the seventh day as mentioned previously (Ishfaq et al., 2019), and 0.1 ml of *E. coli* bacteria (10^9^ CFU/ml) was injected intraperitoneally (Xiao et al., 2014) at day 10.
3. *Mycoplasma gallisepticum* group (C): The *MG* infection model was constructed by left caudal thoracic air sac inoculation with 0.2 ml of *MG* R_*low*_ strain (1 × 10^9^ CCU/ml) at the seventh day as stated above.
4. *Escherichia coli* group (D): 0.1 ml of *E. coli* was injected at a dose of 10^9^ CFU/ml intraperitoneally at day 10.

At the 13th day, 20 chickens from each group were euthanized using the method of cardiac blood collection. Lung samples in each group were collected for further analysis. The protocol for this experiment was approved by the ethics committee at the Harbin Veterinary Research Institute of the Chinese Academy of Agricultural Sciences (SYXK (Hei) 2012-2067).

### Microscopic Lung Examination

The lung tissues were fixed with 10% formalin, dehydrated, and immersed in transparent samples of wax, then cut into slices (3 μm) and stained with hematoxylin and eosin (H & E). The lung tissues were trimmed into small 1-mm^3^ pieces and fixed overnight in 2.5% glutaraldehyde. They were washed with PBS twice and post-fixed in 1% osmium tetroxide at 4°C for 1 h. Next, the tissues were dehydrated by ethanol series and 100% acetone, and embedded in epoxy resins. The ultrathin sections were stained with uranyl acetate and lead citrate and then observed under a GEM-1200ES transmission electron microscope (JEOL Ltd., Tokyo, Japan).

### Transcriptome Sequencing

RNA extracted by Trizol reagent (Invitrogen Inc., Carlsbad, CA, United States) from lung tissue was utilized to construct the final library (BGISEQ-500 RNA-Seq Library) based on the manufacturer’s instructions. The library was validated on an Agilent Technologies 2100 bioanalyzer. The library was amplified with phi29 to make DNA nanoballs (DNB), which had more than 300 copies of one molecule. The DNBs were loaded into the patterned nanoarray, and single-end 50 base reads were generated using sequencing by synthesis. A total of 12 samples were tested using the BGISEQ-500 platform, with an average yield of 21.89 M data per sample. The average alignment ratio of the sample alignment genome was 91.44%, and the average alignment rate of the comparison gene set was 73.29%. Whole transcriptome sequencing data were filtered and mapped to the Chicken genome (Gallus genome Version 5.0.1 NCBI). We used Bowtie2 to compare clean reads to gene sequences and then used RSEM to calculate the gene expression levels for each sample (Li and Dewey, 2011; Langmead and Salzberg, 2012). The DEG-seq method was based on a Poisson distribution (Fold Change > 2 and Adjusted *P*-value < 0.001) (Wang L. et al., 2010; Langmead and Salzberg, 2012). The GEO accession number is GSE130015.

According to the GO/KEGG annotation results and the official classification, we separately classified the functional and biological pathways of the differential genes and used the phyper function in R software for enrichment analysis (Li and Dewey, 2011). Then FDR correction is performed on the *P*-value, and the function of FDR < 0.01 is regarded as significant enrichment (Anders and Huber, 2010).

### Quantitative Real-Time PCR Analysis

The lung tissue samples were homogenized for 2 min at a low frequency of 65 Hz using an automatic tissue homogenizer machine (Shanghai Jingxin Industrial Development Co., Ltd.). Total RNA was extracted using Trizol reagent (Invitrogen Inc., Carlsbad, CA, United States), and the reverse transcription of cDNA was performed according to the manufacturer’s instructions [Takara Biomedical Technology (Beijing) Co., Ltd.]. Quantitative Real-Time PCR (qRT-PCR) was performed to analyze gene expression using a LightCycler96 (Roche, Basel, Switzerland). Each sample was analyzed in triplicate. The fold change in gene expression was calculated using the △△cycle time (Ct) method after the expression level had been normalized with the GAPDH gene taken as the internal standard. The primer sequences are shown in Table 1.

**TABLE 1:** Primers used in QRT-PCR analysis of IL-17 related genes.

**Name**	**Sense strand/sense primer (5′–3′)**	**Antisense strand/antisense primer (5′–3′)**	**Primers origin**
IL-17A	CCATTCCAGGTGCGTGAACT	TTTCTTCTCCAGGCGGTACG	Li et al., 2018
IL-17B	AAGCCAAGGATGAAAGCAGA	CATTGGAGTGTAGGGGTCATC	Pandey et al., 2013
IL-17C	CGAGGACGAGGACCGCTACC	CACGGATGTAATCCACGTCGAAGG	XM_003641945.4
IL-17F	TGAAGACTGCCTGAACCA	AGAGACCGATTCCTGATGT	Kim et al., 2012
CIKS	GCCGTGGTCAGAATATACCGATCC	GTCCTCAGGAGCATCATCCAAGC	XM_025148963.1
TRAF6	CACAGAGGAGACGCAGGGATA	AACAGATCGGGCACTCGTATTT	Rajput et al., 2017
AP-1	AAGCAGAGATGATGCACTGGAAGC	TGGATGTGATGCTGGTGTTGGATG	XM_015296163.1
CXCL1	TGGCTCTTCTCCTGATCTCAATG	GCACTGGCATCGGAGTTCA	Wu et al., 2010
CXCL2	GCCCTCCTCCTGGTTTCAG	TGGCACCGCAGCTCATT	Wu et al., 2010
CXCL8	CCAAGCACACCTCTCTTCCA	GCAAGGTAGGACGCTGGTAA	Shojadoost et al., 2015
GMCSF	CCGTTTCAGGAACCAGAGAG	GTCTGGCTGCTGGACATTTT	Lim et al., 2012
MUC5AC	AAGACGGCATTTATTTCTCCAC	TCATTACCAACAAGCCAGTGA	Fan et al., 2015
MMP1	ATTTGATGCCATTACCACTT	ACTTCATCCCTTTCAATGTTCT	Zhu et al., 2014
MMP3	ATCAGGCTCTACAGTGGTG	ATGGGATACATCAAGGCAC	Zhu et al., 2014
C/EBPβ	ATTACGAGGCGGACTGTTTGG	CGGGTGAGGCTGATGTAGGTG	Fu et al., 2014
NF-κB	AGAAAAGCTGGGTCTTGGCA	CCATCTGTGTCAAAGCAGCG	Hoang et al., 2017

### Measurement of Cytokines and Chemokines by ELISA

Six lung tissue samples from each group were extracted for analysis with enzyme-linked immunosorbent assay (ELISA) kits in accordance with the manufacturer’s instructions (Beijing Cheng Lin Biological Technology Co., Ltd.). Five cytokines and chemokine activities were detected including CXCL1, CXCL2, MMP1, GM-CSF, and MUC5AC. Lung tissue samples were cut and weighed (100 mg). 500 μL of cold PBS (PH 7.4) was added, and the mixture was homogenized for 2 min at a low frequency of 65 Hz using an automatic tissue homogenizer machine (Shanghai Jingxin Industrial Development Co., Ltd.) followed by centrifugation (3,000 × g for 20 min) at 4°C. The supernatant was collected, and the samples were loaded on a 96-well microtiter plate in duplicate along with a blank control sample. The readings were taken on an iMARKTM microplate reader (Bio-Rad Co., Ltd. Shanghai, China) at a wavelength of 450 nm.

### Western Blot

Western blotting was used to measure the IL-17 pathway-related target proteins. The total proteins of lung tissue were extracted by whole-cell lysis assay, and the supernatant protein content was determined using a BCA protein assay kit (Wanlei, Liaoning, China). The membranes were incubated overnight on a shaker at 4°C with primary antibodies against β-actin (1:5000 dilution), TRAF6 (1:500 dilution), CIKS (Act1), NFκB (p65), AP1 (c-jun), and C/EBPβ (1:1000 dilution). All the primary antibodies were purchased from Bioss Bioscience Inc., Beijing, China. Secondary anti-mouse and anti-rabbit IgG peroxidase was used for 1 h, and then bound immune complexes were detected using enhanced chemiluminescence (ECL) detection. The protein bands were analyzed by densitometry using Image J (Ver 1.42, National Institutes of Health, United States).

### Statistical Analysis

The results are presented as mean ± standard deviation (SD). The significance was determined using one-way ANOVA followed by LSD and Dunnett’s T3 test. The data were analyzed using GraphPad Prism (version 5.01). Values with *p* < 0.05 were considered statistically significant. Heatmaps were created with Heatmap Illustrator (1.03.7). Volcano maps, column charts, bubble charts, and cluster thermal maps were all created using the BGI data mining website^1^.

## Results

### Pathological Changes

**FIGURE 1:**
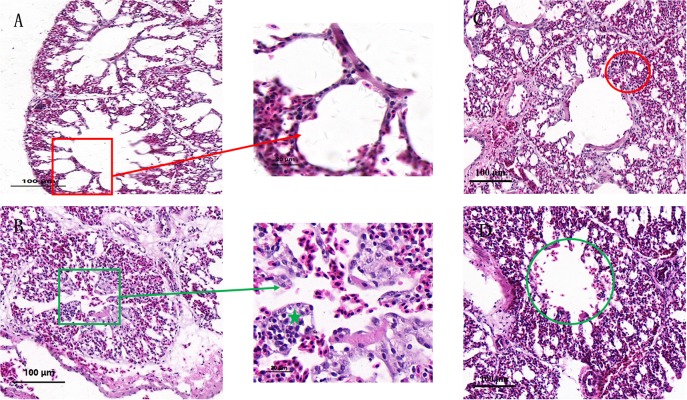
Effects of co-infection on the histology of chicken lung. Paraffin sections of tracheal tissues from the control group **(A)**, co-infection group **(B)**, *MG* group **(C)**, and *E. coli* group **(D)**, stained with hematoxylin-eosin (100×). **(A)** Red frame shows normal bronchioles and alveolar structures. **(B)** Green frame shows bronchial epithelial adhesions, epithelial cells degeneration, necrosis. Green star illustrates lymphocyte infiltrations around the bronchi. **(C)** Red circle shows intersomese hyperplasia in the MG group. **(D)** Green circle indicates slight bleeding in the tube cavity.

Histopathological examination (Figure 1) was performed in order to understand the effects of co-infection on chicken lung better. In the control group (Figure 1A), the bronchial monolayer ciliated columnar epithelium was intact, and there was no abnormality in the alveolar cavity. Bronchial epithelial cell adhesions and epithelial cell degeneration were observed in the co-infection group (Figure 1B) compared to the control group. Lymphocyte infiltration mainly occurred around the bronchi region, and the alveolar space was reduced in the *MG* group (Figure 1C). There were symptoms of mild interstitial pneumonia in the MG-infection group, and slight bleeding in the bronchial tube cavity was observed in the *E. coli*-infection group (Figure 1D).

### Ultrastructural Changes

**FIGURE 2:**
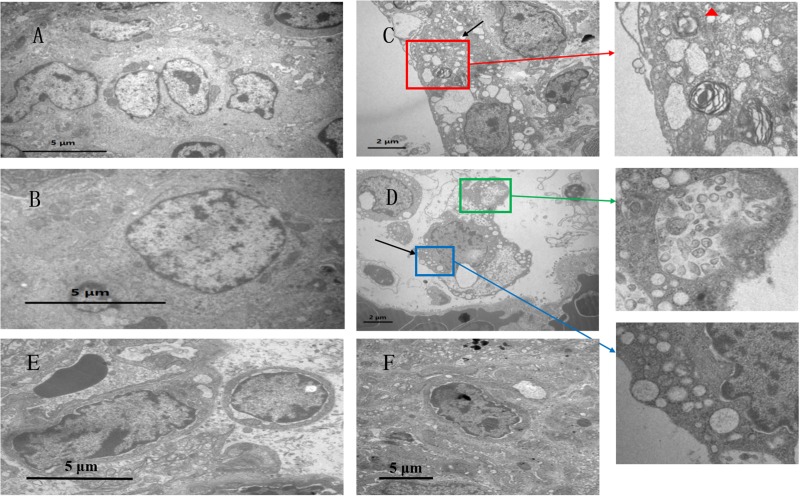
Ultrastuctural analysis showing the effects of co-infection on chicken lung. Transmission electron microscopy of **(A,B)** control group, at a magnification of 8,000×. Panel **(C,D)** belong to the co-infection group, at a magnification of 15,000×. **(C)** Red frame shows that alveolar type II cells are damaged, indicating cytoplasmic vacuolar degeneration. Red triangle shows that eosinophilic lamellar bodies are empty. **(D)** Blue frame shows that Clara cells have severe vacuolization. Green frame shows that Clara particles are small, and the structure is ambiguous. In addition, Clara particles have low electron density. **(E)** The MG group shows minor vasotec in alveoid type I cells. **(F)** In the *E. coli* group, the nucleus is separated from the substrate membrane, and the distribution of nuclear kernels is abnormal.

Figure 2 shows the ultrastructure of chicken lungs. In the control group (Figures 2A,B), the alveolar epithelial cells were structurally intact, and the adjacent cells were tightly connected. The adjacent cells were oval in shape and had microvilli on their surfaces. Capillary congestion was observed in the co-infection group compared to the control group. Alveolar type II cells were damaged (Figure 2C), cytoplasmic vacuolar degeneration was severe, and eosinophilic lamellar bodies were empty in the co-infection group. At the same time, type II epithelial cells proliferated markedly, occurring as beads along the alveolar wall or in piles. Clara cells also had severe vacuolization and degeneration, and the cytoplasm was even completely shed in the co-infection group (Figure 2D). Compared to the co-infection group, there were no significant lesions in the *MG* infection group (Figure 2E) and *E. coli* group (Figure 2F).

### Global DEGs Among Infected Chickens and GO Function

**FIGURE 3:**
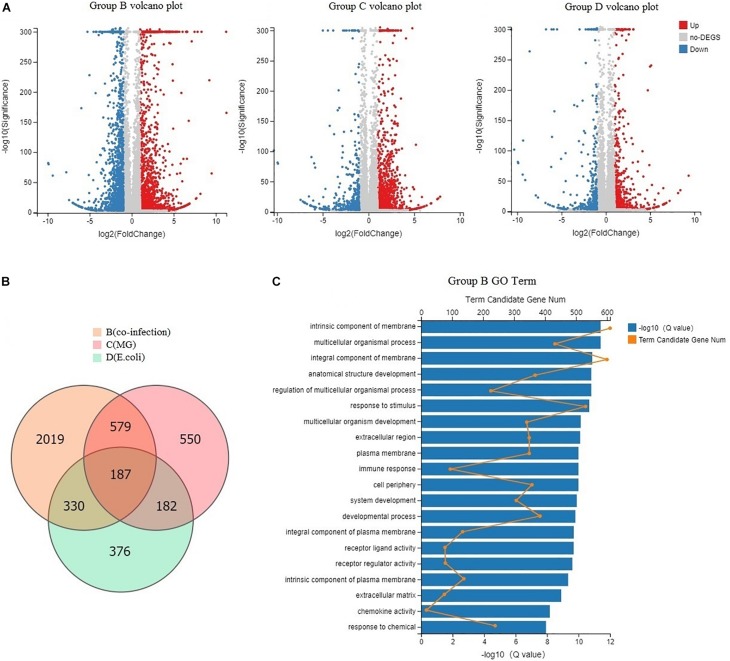
Global DEGs among infected chickens and GO function. **(A)** Volcano plots displaying DEGs in the three infected groups compared to the control group. Red dots (Up) represent significantly up-regulated genes; blue dots (Down) represent significantly down-regulated genes; gray dots (No) represent insignificantly DEGs (Fold Change > 2 and Adjusted *P*-value < 0.001). **(B)** Venn diagram of the three infected groups; orange represents co-infection, light pink represents *MG* infection, and blue represents *E. coli* infection. **(C)** GO categorization (biological process, cellular component, and molecular function) of the unigenes in the transcriptome of chicken lung induced by co-infection. The length of the *X*-axis column represents the size of the *Q*-value. The value of the point on the fold line above *X* is the number of differential genes annotated to the GO Term.

Sampling directly from the lung yielded sufficient quantities of RNA to assess transcripts from each chicken and map to 18,043 Gallus gallus genes. We found 3,115 DEGs between the co-infection group and the control group, 1,498 DEGs between the *MG* group and the control, and 1,075 DEGs between the *E. coli* group and the control (Figure 3A). The results indicated that the host mounts a rapid response to co-infection and that the number of DEGs is far greater than in the individual infections. From the Venn diagram, we found that 2,019 DEGs were only in the co-infection group and that 1,096 DEGs were shared by the three infected groups (Figure 3B).

Annotation through GO analysis mainly assigned the DEGs to biological process, cellular component, and molecular functions. In Figure 3C, we show the degree of enrichment GO term in a histogram format and plot the top 20 GO terms with the smallest Q values, including response to stimulus, immune response, chemokine activity, and so on.

### Pathway Analysis

**FIGURE 4:**
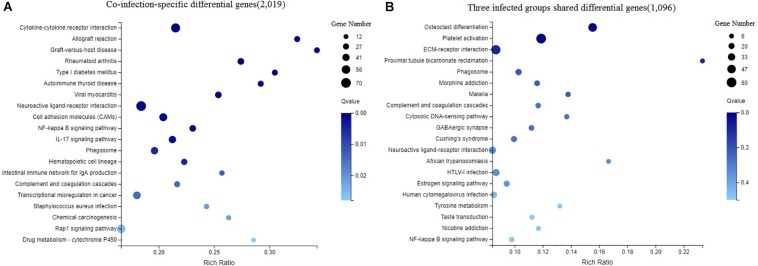
Enriched bubble chart showing the enrichment of the KEGG Pathway in three dimensions. *X*-axis is enrichment ratio, *Y*-axis is KEGG Pathway. Bubble size indicates the number of genes annotated to a KEGG Pathway. Color represents the enriched *Q*-value. The darker the color, the smaller the *Q*-value. **(A)** The top 20 KEGG enrichment results of co-infection-specific differential genes (2,019). **(B)** The top 20 KEGG enrichment results of the differential genes shared by the three infected groups (1,096).

**FIGURE 5:**
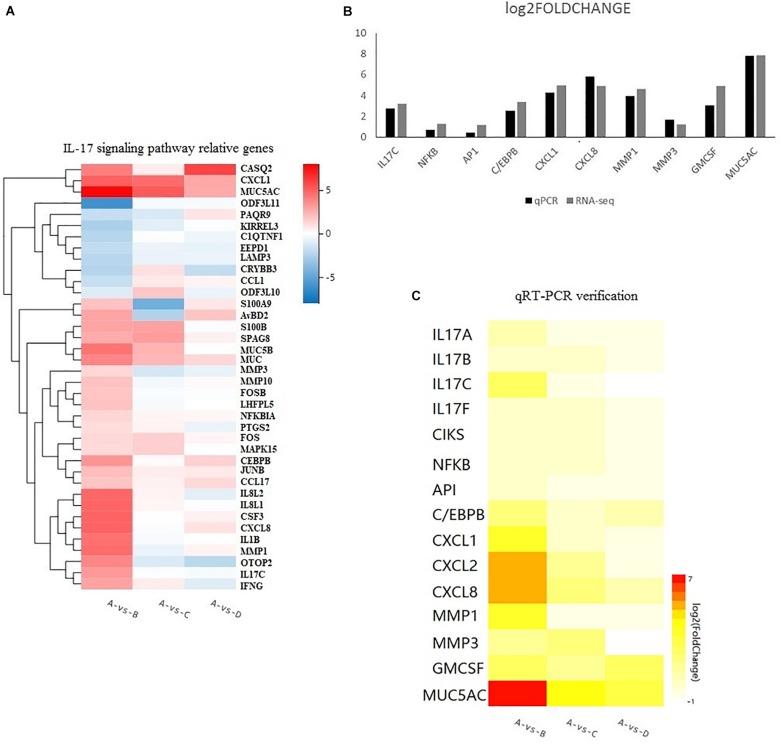
**(A)** Heatmap showing the relative gene expression of 38 genes responsible for the IL-17 signaling pathway according to the results of RNA-seq. The horizontal axis represents log2 fold change. The vertical axis represents the genes. A bright red color indicates stronger up-regulation and a bright blue color indicates stronger down-regulation in expression. **(B)** Comparison of RNA-seq and qRT-PCR results. Results are expressed as relative fold change. **(C)** The levels of 15 up-regulated genes from qPCR. A bright red indicates stronger up-regulation and a white color indicates no significant change in expression.

The DEGs in chicken from the three infected groups were further annotated by KEGG. Figure 4A shows the top 20 KEGG enrichment results for co-infection-specific differential genes (2,019). Figure 4B shows the top 20 KEGG enrichment results for the differential genes shared by the three infected groups (1,096). The enriched bubble charts show the enrichment of KEGG pathways in three dimensions, which were analyzed with Q-values and the number of genes in the pathway. By comparing the two bubble graphs, we found that the IL-17 signaling pathway is only enriched significantly in the co-infection group, so we chose the IL-17 signaling pathway to carry out differential clustering analysis. The results for the co-infection group show considerable changes in the genes related to the IL-17 pathway, including CXCL1, MMP1, MUC5AC, GM-CSF, etc. (Figure 5A).

### Quantitative RT-PCR Verification

The sequencing data were verified by verifying 10 genes selected from the RNA-Seq expression profiles with the co-infection group via the qRT-PCR method, including upstream (IL-17C, NFκB,AP1, and C/EBPβ) and downstream genes (CXCL1, CXCL8, MMP1, MMP3, GM-CSF, and MUC5AC), as shown in Figure 5B. The sequencing results were confirmed to be reliable. Furthermore, to investigate the IL-17 signaling pathway, we searched for information about the IL-17 pathway in the KEGG database and added five genes (IL-17A, IL-17B, IL-17F, CXCL2, and CIKS) to this pathway. Fifteen genes responsible for the IL-17 pathway were included to produce a heatmap for the relationships between the three infected groups, as shown in Figure 5C. The heatmap shows that there are different degrees of eruption of chemokines and mucins secreted by the three infected groups. The co-infection group showed a significant increase. However, we found that the expression of CXCL8, MMP3, and MUC5AC was higher in the *MG*-infection group compared to the *E. coli*-infection group.

### Influence of Infected Groups on Cytokines and Chemokines

**FIGURE 6:**
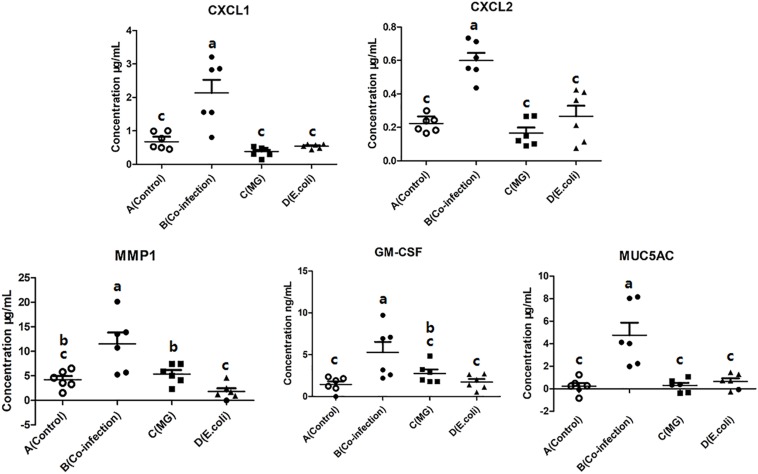
Scatter plot of inflammatory mediators (CXCL1, CXCL2, MMP1, GM-CSF, and MUC5AC) detected by ELISA in lung tissues. Bars represent the mean ± SD. Bars with different superscript letters are significantly different (0.01 < *P* < 0.05).

The influence of the three infected groups was determined by measuring the concentrations of CXCL1, CXCL2, MMP1, GM-CSF, and MUC5AC in the lungs by using a sandwich ELISA. The results showed that the cytokine and chemokine activities were significantly (*p* < 0.05) enhanced in the co-infection group (Figure 6) compared to the control group. However, there was no significant increase (*p* > 0.05) in the *MG* group and the *E. coli* group compared to the control group.

### Western Blot of IL-17 Pathway-Related Target Proteins

**FIGURE 7:**
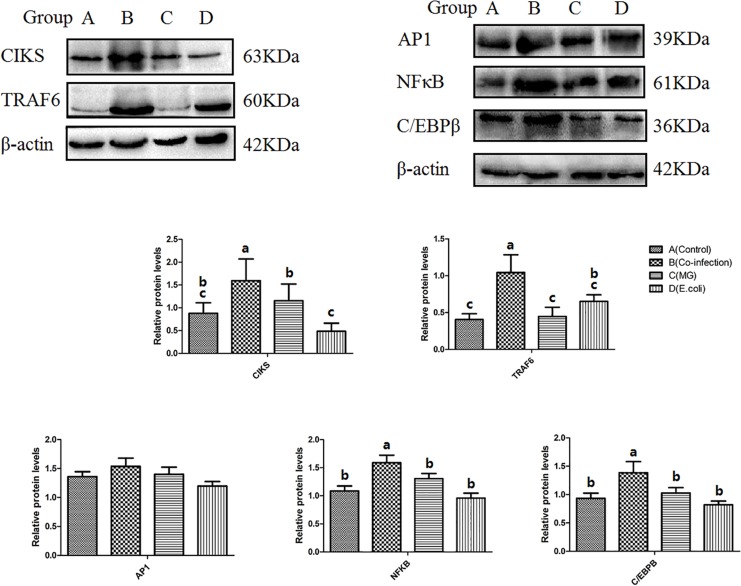
Protein levels of CIKS, TRAF6, NFκB, AP1, and C/EBPβ as measured by Western blot. β-actin was used as a control. Bars represent the mean ± SD. Bars with different superscript letters are significantly different (0.01 < *P* < 0.05).

We selected five key proteins in the IL-17 signaling pathway for WB experiments in order to conduct in-depth research, namely CIKS, TRAF6, NFκB, AP1, and C/EBPβ. From the results for these five proteins (Figure 7), we found that two (CIKS and TRAF6) of the pathways are significantly different in the three infected groups. The protein expressions of CIKS and TRAF6 were significantly (*p* < 0.05) upregulated in the co-infection group compared to the *MG* and *E. coli* groups. Of the remaining three parallel levels of protein, C/EBPβ and NFκB significantly (*p* < 0.05) increased in the co-infection group compared to the control group and the mono-infected groups. Meanwhile, AP1 showed no significant difference among the three infected groups. See Supplementary Material for original blots.

## Discussion

The mechanisms by which viruses promote secondary bacterial co-infection are diverse throughout the airway (Bakaletz, 2017). In this study, transcriptomic sequencing identified genes and pathways responsive to the infection in chicken lung. There were 3,115/1,498/1,075 DEGs among the three infection groups compared to the control group, respectively, meeting the criteria of FOLDCHANGE > 2 and FDR < 0.001. QPCR analysis showed that the expression trend of 10 genes between the co-infection and control groups (IL-17C, NFκB, AP1, C/EBPβ, CXCL1, CXCL8, MMP1, MMP3, GM-CSF, and MUC5AC) was consistent with the RNA-seq results. The results of RNA-seq in our study of the *MG* group are similar to those reported from in previous research (Beaudet et al., 2017). All of these findings indicated that the RNA-seq data were reliable.

Through comparative transcriptomic analysis to produce a Venn diagram, we found that there were far more differential genes than in the other two infection groups. We divided these differential genes into two categories to analyze the special features of co-infection and individual infections. One was DEGs that were unique in the co-infection group (2,019), and the nother was DEGs that were shared by the three infection groups (1,096). By comparing the KEGG enrichment results of the two categories of DEGs, we found that the IL-17 signaling pathway was significantly enriched in the co-infection group. Through research on IL-17 in recent years, it has been found that IL-17 mediates the production of many inflammatory cytokines and chemokines, including CXCL1, GM-CSF, MMPs, TNFα, IL-8, and so on Kurai et al. (2013), Amatya et al. (2017), Brown et al. (2017), Nadeem et al. (2018). Hence, we focus on this pathway among the three infection groups.

In this study, we selected 38 genes that are differentially expressed in the IL-17 pathway from the results of RNA-seq and compared the expression of these genes among the three infected groups. For further study, the qPCR method was used to analyze 15 target genes (IL-17A, IL-17B, IL-17C, IL-17F, CIKS, NFκB, AP1, C/EBPβ, CXCL1, CXCL2, CXCL8, MMP1, MMP3, GM-CSF, and MUC5AC) in this pathway. The increase in the IL-17C gene was markedly significant among the four IL-17-related cytokines. Research by Jungnickel shows that IL-17C promotes neutrophilic inflammation in the tumor microenvironment and suggests that IL-17C links a pathologic microbiota, with enhanced tumor growth (Jungnickel et al., 2017). RNA-seq results showing that hydrogen sulfide exposure triggers chicken trachea inflammatory injury also show that IL-17C is upregulated by 19.8-fold (Chen et al., 2019). Our research indicated that IL-17C as one of the IL-17-related cytokines and behaves actively in the lung co-infection model. Nevertheless, more detailed studies are needed to investigate the cellular source of IL-17 in chicken lung and to determine which cell population has the IL-17 signaling pathway upregulated upon MG and E.coli infection and whether the silencing of this pathway could reduce the severity of infection.

Furthermore, we selected five key upstream target proteins (CIKS, TRAF6, NFκB, AP1, and C/EBPβ) in the IL-17 pathway and detected their expression by WB. CIKS (ACT1, TRAF3IP2), an IL-17-receptor-complex adaptor, binds and stabilizes mRNAs encoding key inflammatory proteins (Herjan et al., 2018). Mechanistically, the interaction between CIKS and TRAFs (tumor necrosis factor receptor-associated factor proteins) is essential in the IL-17 pathway (Qiu et al., 2016). The present study has pointed out that p300-dependent histone H3 acetylation and C/EBPβ-regulated IKKβ expression contribute to thrombin-induced IL-8/CXCL8 expression in human lung epithelial cells (Huang et al., 2017). In the absence of NF-κB signaling, proteasomal degradation of C/EBPβ is increased by a JNK-independent mechanism and promotes death from TNF-α (Wang Y. et al., 2010), which suggests that we can use C/EBPβ as a target protein for the treatment of IL-17 outbreaks on co-infection.

Related inflammatory mediators and chemokines were detected by the ELISA method, including CXCL1, CXCL2, MMP1, GM-CSF, and MUC5AC. Combined with the results of qPCR and microscopic examination, we found that co-infection enhances the production of inflammatory mediators and inflammatory injury. Previous research has shown that IL-17C functions in an autocrine/paracrine manner to increase the production of epithelial CXCL1 but that CXCL1 is associated with the collection and inflammation of neutrophils (Jamieson et al., 2019). Since these chemokines play a crucial role in lung invasion and inflammation (Nawrocki-Raby et al., 2003; Brown et al., 2017; Naveed et al., 2017), we define them as one of the evaluation indicators. The most significant difference is MUC5AC, which may constitute a key component of sputum production and mediate disease severity (Kesimer et al., 2017). The results also illustrate the importance of IL-17 in co-infection and the damage caused by chemokines to lung tissue.

From the results regarding pathological and ultrastructural changes among the four groups, we found that co-infection causes severe interstitial pneumonia symptoms that disrupt the structure of the bronchioles in the lungs. The results of transmission electron microscopy showed that alveolar epithelial cells and Clara cells are severely vacuolated and that cytoplasm even completely falls off. Clara cells have multiple lung-protection functions, contributing anti-microbial peptides, several cytokines and chemokines, and mucins that infiltrate into the extracellular fluid lining the airspaces (Reynolds and Malkinson, 2010). The published study also found that the epithelium is a target for factors released by infiltrating inflammatory cells. In airway inflammatory diseases such as asthma, cilia loss and excessive mucus secretion are major pathological features (Del Donno et al., 2000; Kesimer et al., 2013). Hence, mucins expand their role in innate lung protection and provide new molecular targets for gene therapy.

## Conclusion

In summary, our study demonstrates that co-infection with *MG* and *E. coli* could induce more severe inflammatory injury than in individual infection, bronchial cilia loss, and mucus accumulation in the lungs of chickens. The *in vivo* experiments show that co-infection causes the activation of the IL-17 signaling pathway and that the most abundant target proteins are IL-17C and C/EBPβ, resulting in the production of numerous inflammatory factors and chemokines. This study provides insight into the molecular mechanisms of co-infection with *MG* and *E. coli* and reveals target proteins for targeted drug therapy.

## Data Availability Statement

The datasets analyzed in this manuscript are not publicly available. Requests to access the datasets should be directed to GEO, accession number is GSE130015.

## Ethics Statement

The protocol for this experiment was approved by the ethics committee at the Harbin Veterinary Research Institute of the Chinese Academy of Agricultural Sciences [SYXK (Hei) 2012-2067]. Samples were only collected from animals for laboratory analyses, avoiding unnecessary pain and suffering of the animals. The studies did not involve endangered or protected species.

## Author Contributions

ZW, MI, and JL designed the study. ZW, JB, YL, JW, QZ, RL, and LD performed and collected data from experiments and analyzed the data. ZW and MI wrote the manuscript. All authors read and approved the final manuscript.

## Conflict of Interest

The authors declare that the research was conducted in the absence of any commercial or financial relationships that could be construed as a potential conflict of interest.

## Footnotes

1 http://report.bgi.com

## Abbreviations

AP1: transcription factor AP-1
C/EBP β: CCAAT enhancer binding protein beta
CIKS: adapter protein CIKS
CRD: chronic respiratory disease
CXCL1: chemokine (C-X-C motif) ligand 1
CXCL2: chemokine (C-X-C motif) ligand 2
DEGs: differentially expressed genes
*E. coli*: *Escherichia coli*
FDR: false discovery rates
GM-CSF: granulocyte-macrophage colony-stimulating factor
GO: gene ontology
IL-17: interleukin 17
KEGG: Kyoto Encyclopedia of Genes and Genomes
*MG*: *Mycoplasma gallisepticum*
MMP1: matrix metalloproteinase-1
MUC5AC: mucin 5 subtype AC
NF κ B: nuclear factor NF kappa B p65 subunit
TRAF6: tumor necrosis factor receptor-associated factor protein 6
