# Supplementary figures and images for: Co-infection of Mycoplasma gallisepticum and Escherichia coli Triggers Inflammatory Injury Involving the IL-17 Signaling Pathway
Source: Front Microbiol. 2019 Nov 15;10:2615. doi: 10.3389/fmicb.2019.02615 (PMC6872679; doi:10.3389/fmicb.2019.02615)

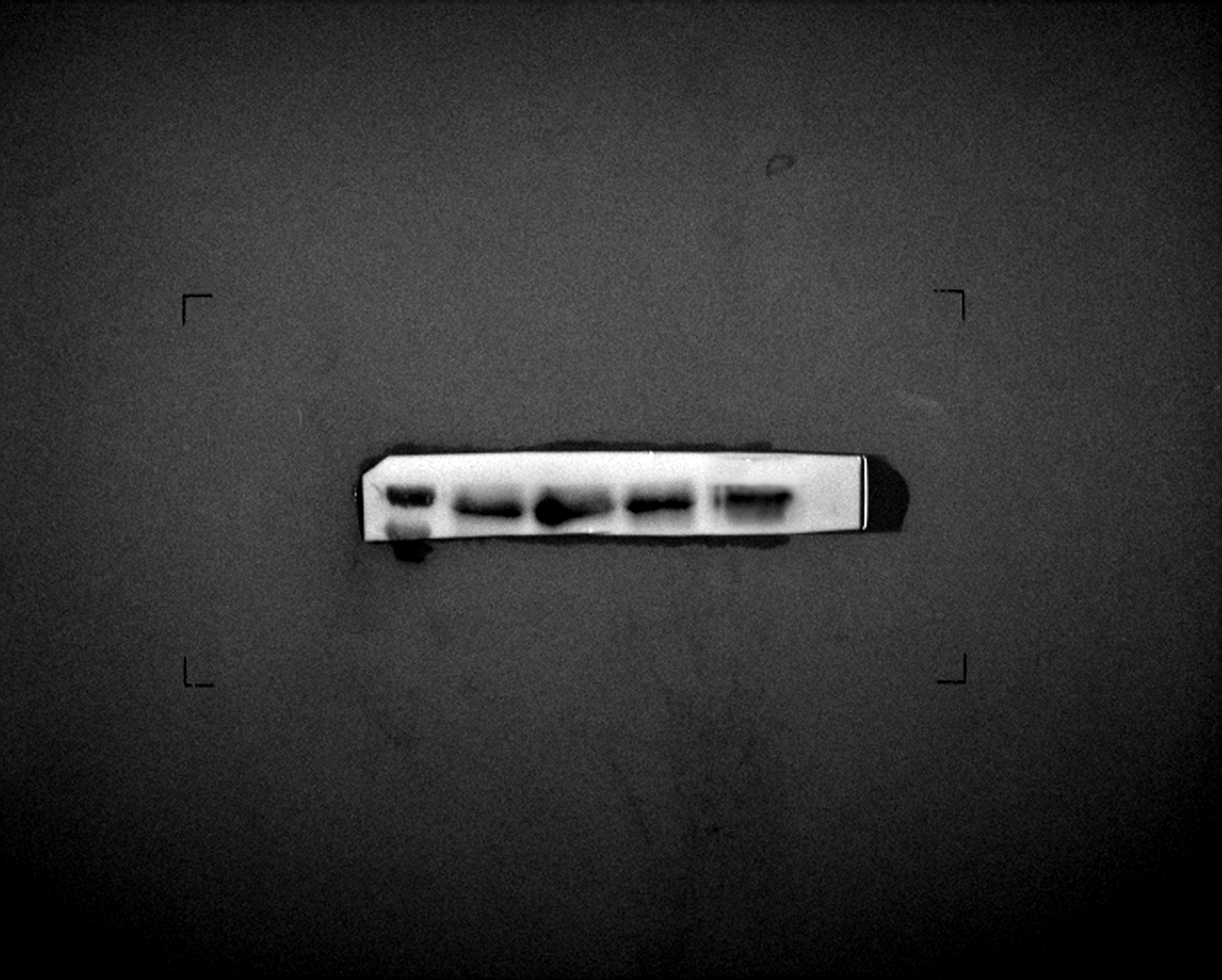

Supplement: Supplementary file 1 [file Data_Sheet_1.ZIP › AP1.tif]

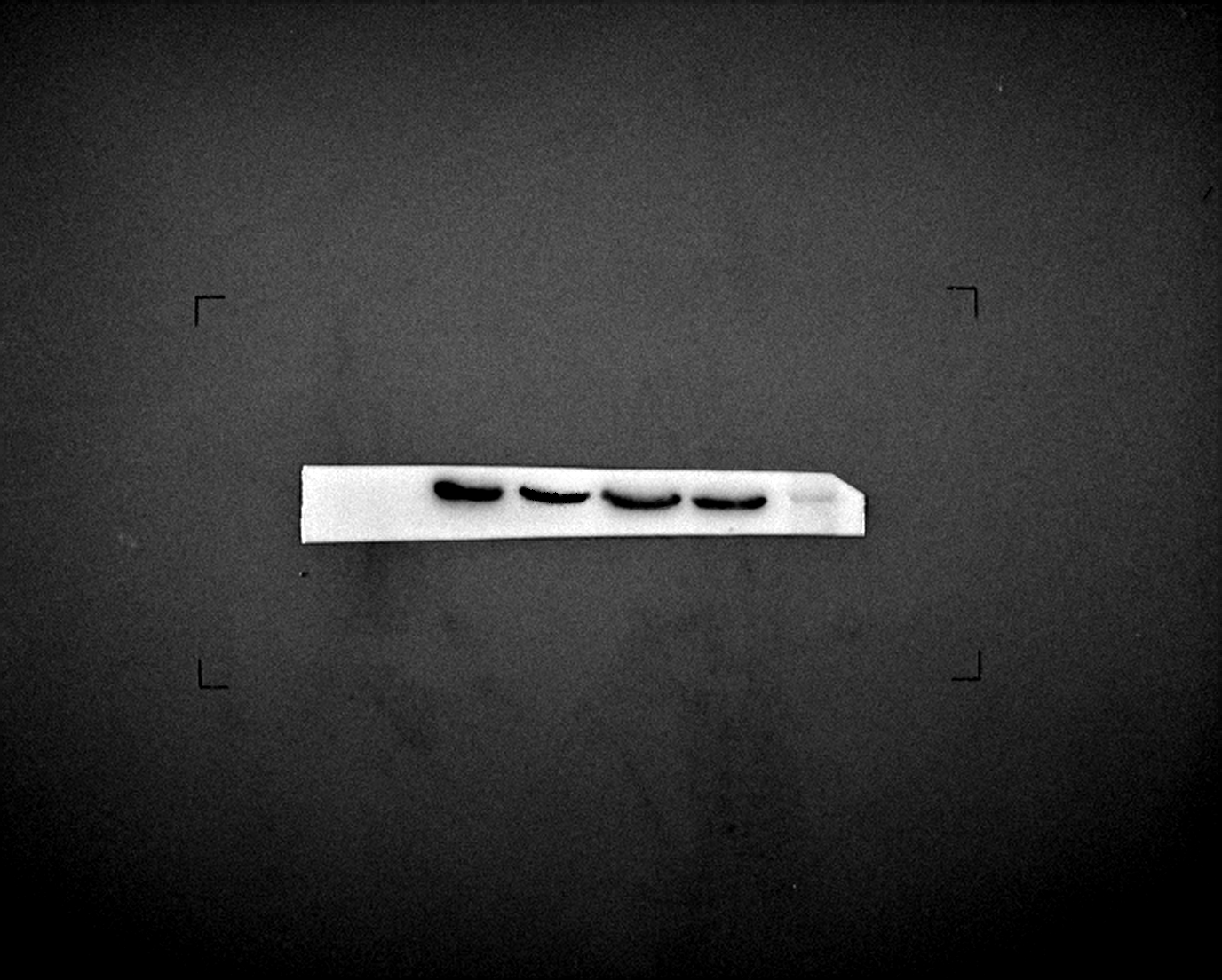

Supplement: Supplementary file 1 [file Data_Sheet_1.ZIP › BACTIN1.tif]

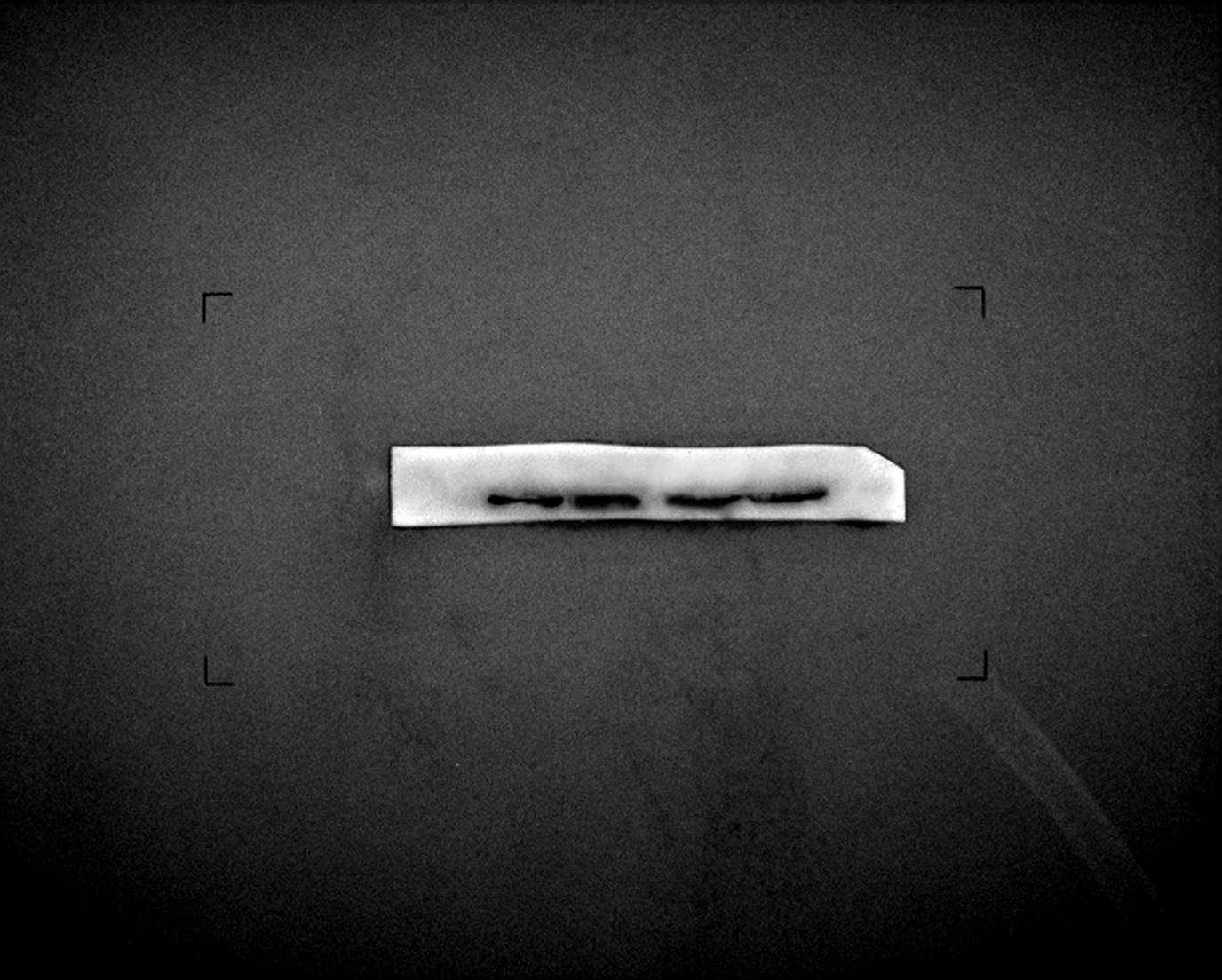

Supplement: Supplementary file 1 [file Data_Sheet_1.ZIP › BACTIN2.tif]

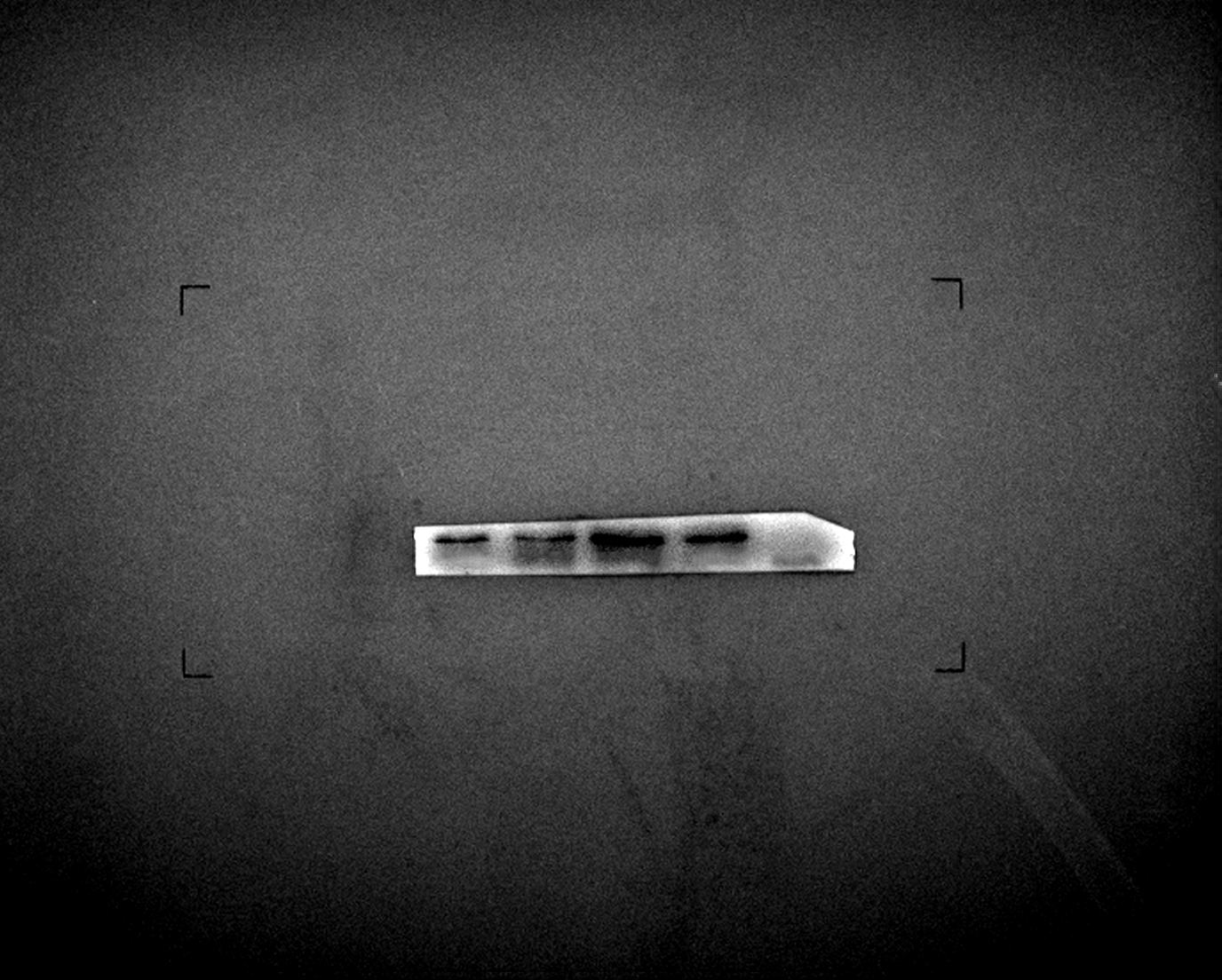

Supplement: Supplementary file 1 [file Data_Sheet_1.ZIP › CEBP.tif]

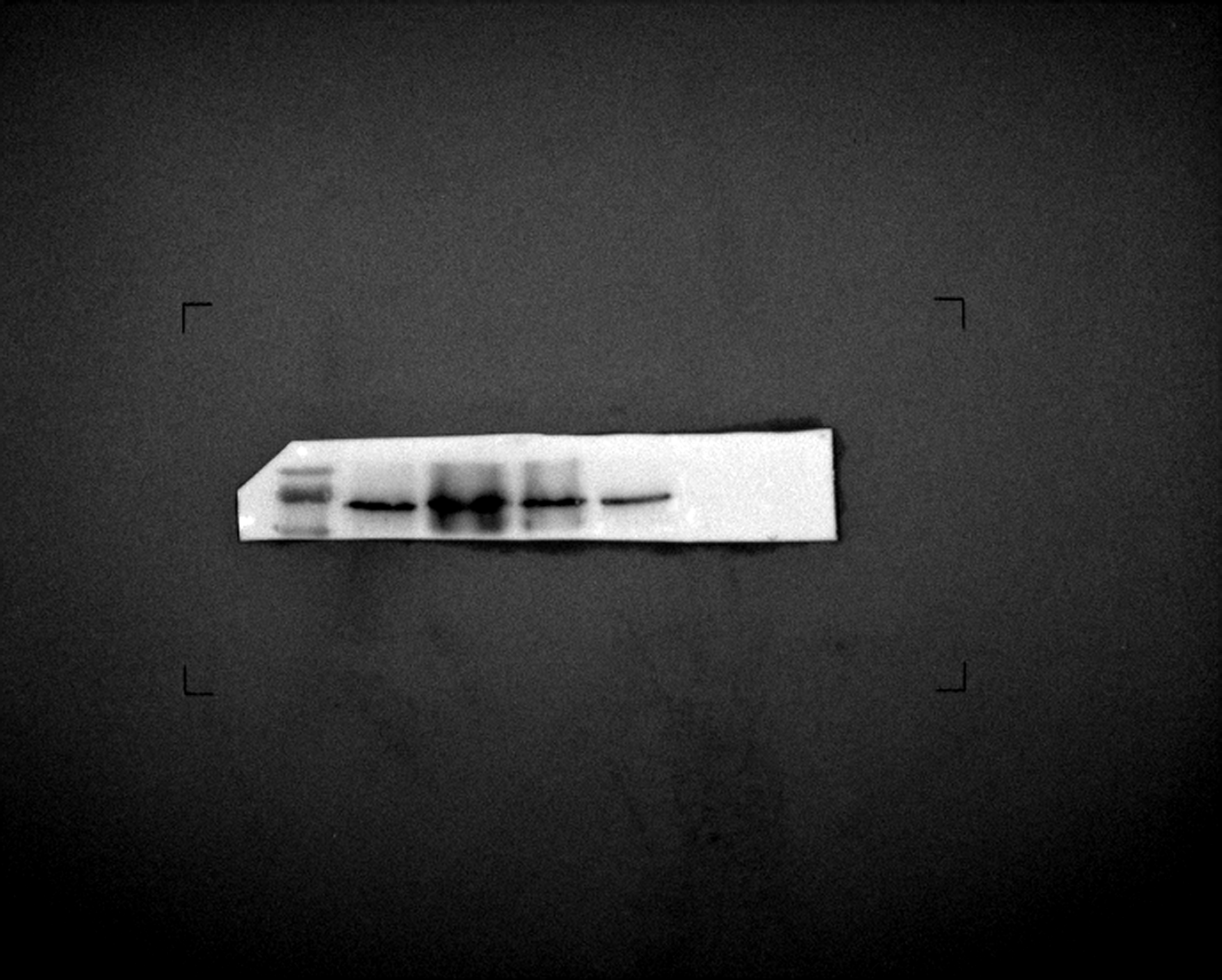

Supplement: Supplementary file 1 [file Data_Sheet_1.ZIP › CIKS.Tif]

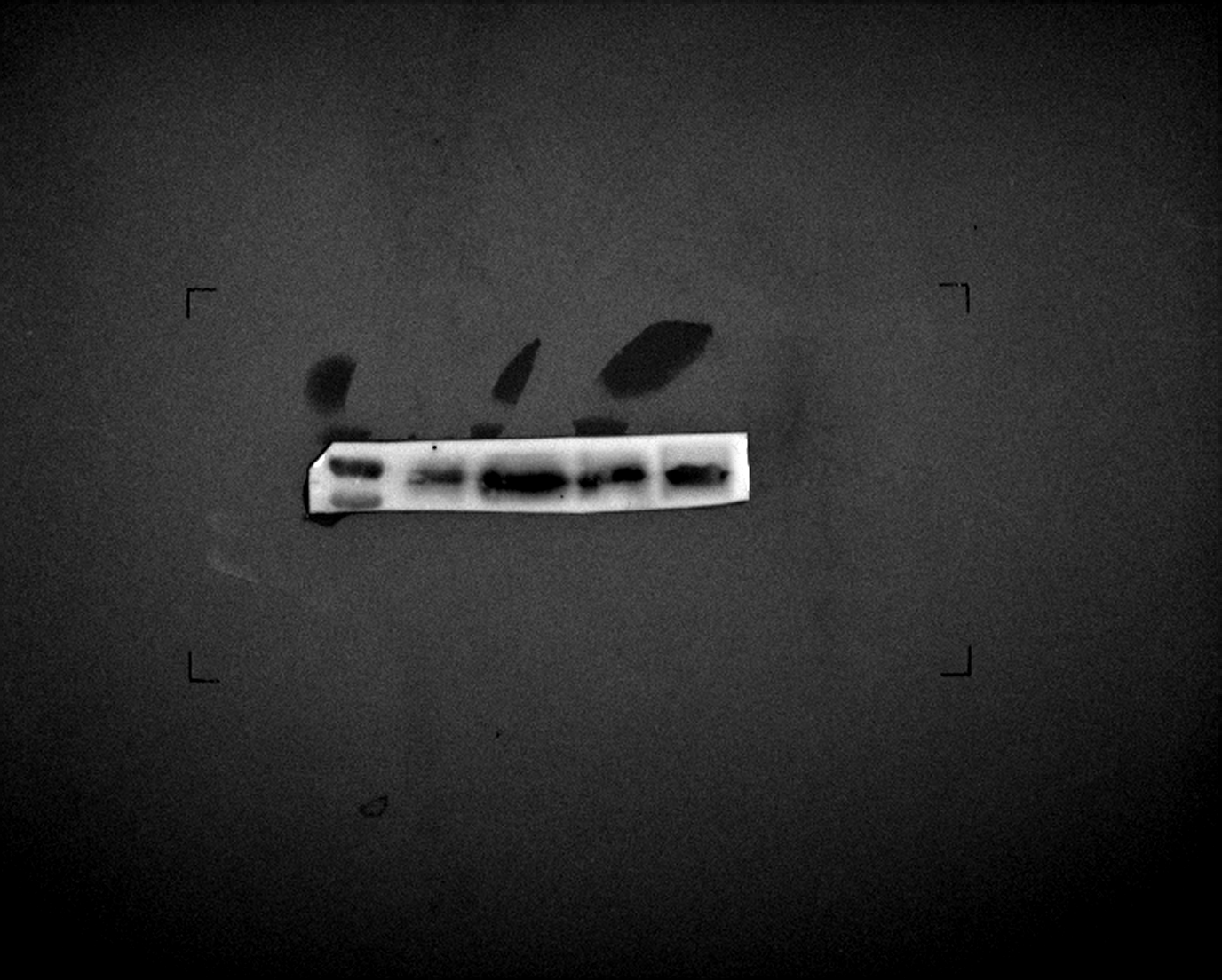

Supplement: Supplementary file 1 [file Data_Sheet_1.ZIP › P65.tif]

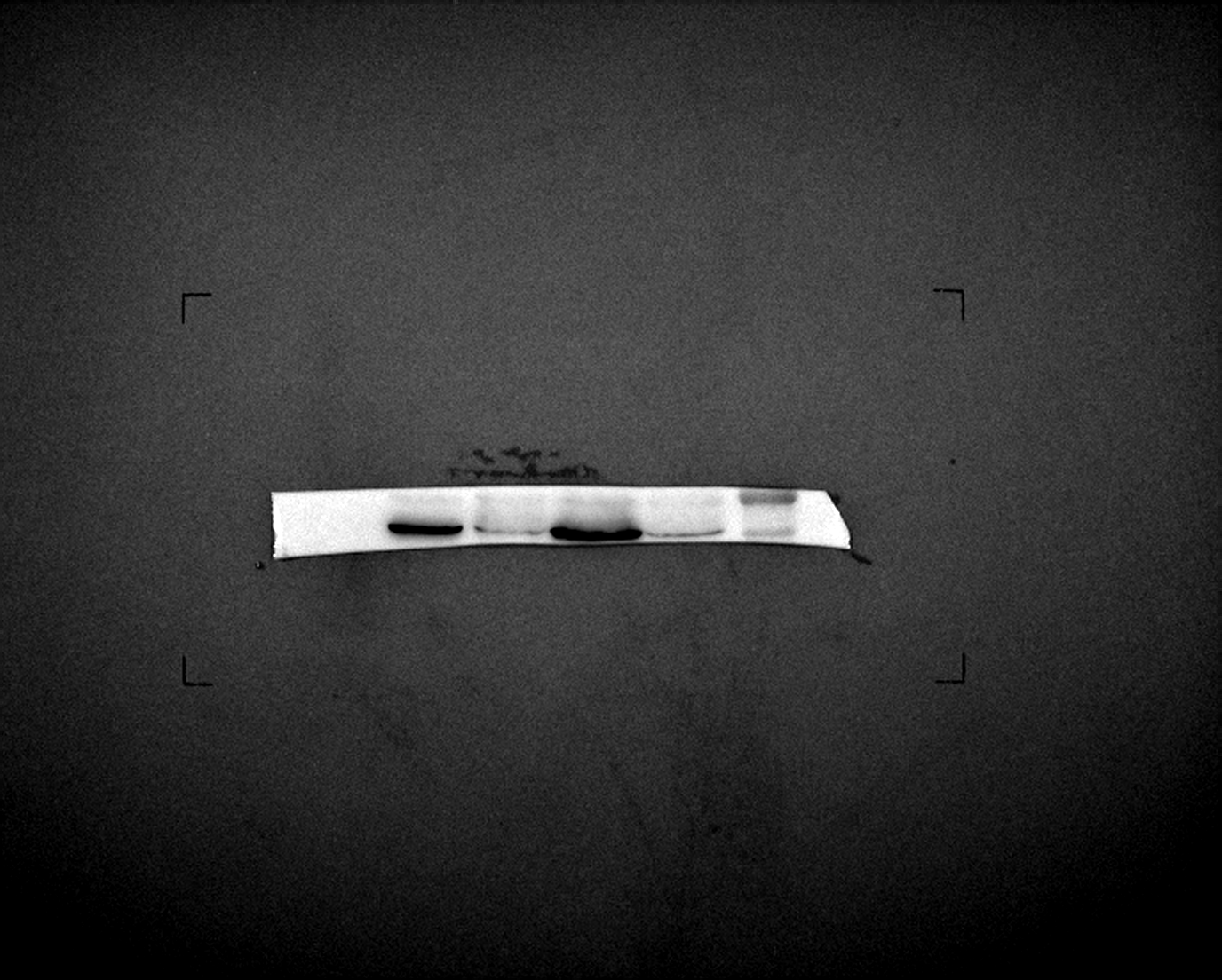

Supplement: Supplementary file 1 [file Data_Sheet_1.ZIP › TRAF6.Tif]

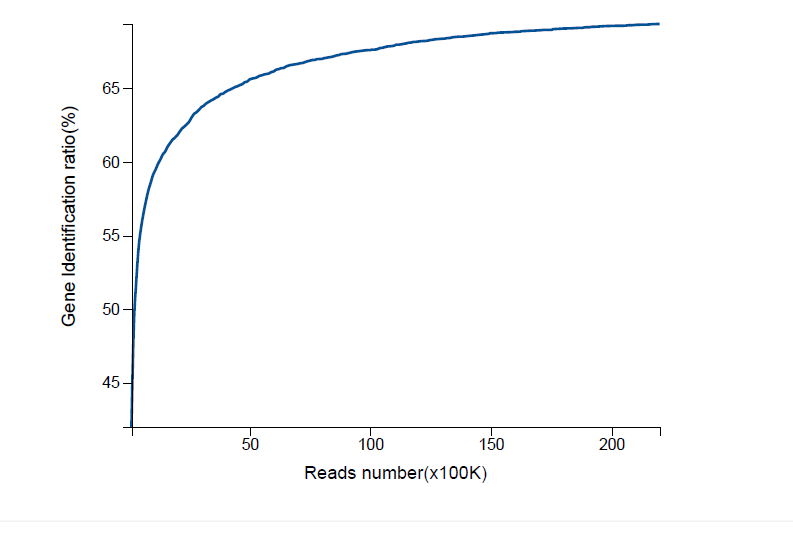

Supplement: Supplementary file 2 [file Image_1.PNG]

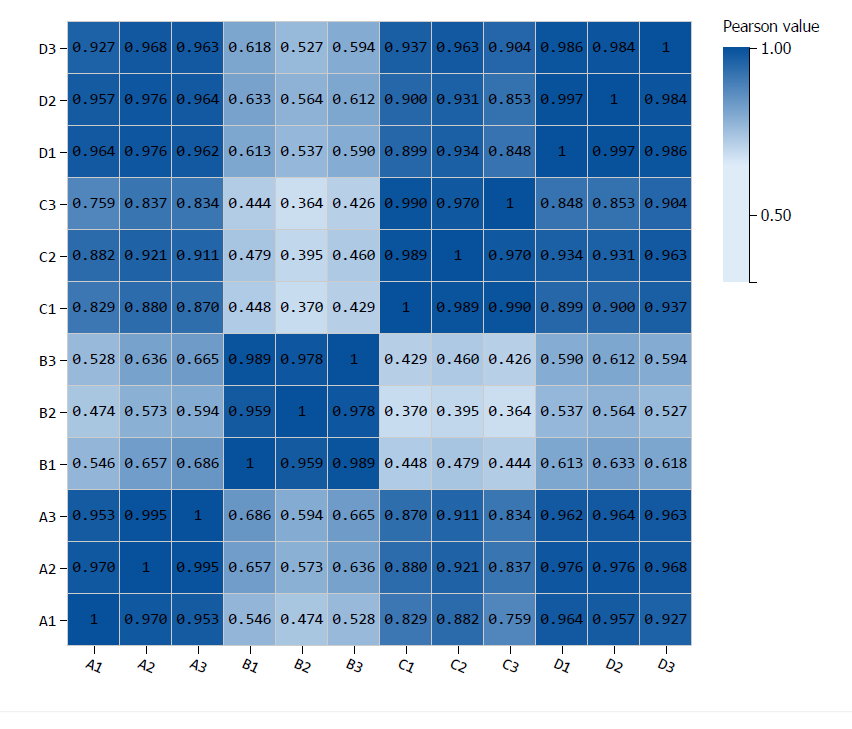

Supplement: Supplementary file 3 [file Image_2.PNG]
